# Selected determinants of anxiety and depression symptoms in adolescents aged 11–15 in relation to the pandemic COVID-19 and the war in Ukraine

**DOI:** 10.3389/fpubh.2024.1480416

**Published:** 2025-01-14

**Authors:** Izabela Grzankowska, Malgorzata Wójtowicz-Szefler, Monika Deja

**Affiliations:** Department of Psychology, Kazimierz Wielki University, Bydgoszcz, Poland

**Keywords:** mental health, intrapsychic factors, social stressors, COVID-19 pandemic, war in Ukraine, adolescents

## Abstract

**Introduction:**

The ongoing COVID-19 pandemic, which began in early 2020, and the outbreak of war in Ukraine in 2022 (a country bordering Poland on the east) have significantly impacted the mental health of young people in Poland, leading to increased rates of depression, anxiety, and other mental health issues. The rising number of individuals struggling to cope with daily stressors, as well as non-normative stressors, may indicate a decrease in the individual’s potential, specifically in skills, attitudes, and competencies required to overcome difficulties that they encounter. It can be assumed that for young people, maintaining mental health under the influence of social stressors, such as the pandemic and the ongoing war in Ukraine, depends on the ability to adapt positively, which is the ability of young individuals to adjust to situational demands in a way that allows them to effectively manage those situations. The aim of the conducted study was to determine whether social stressors, namely the pandemic and the war in Ukraine, and intrapsychic factors such as beliefs about oneself, others, life, and the world, influence the occurrence of anxiety and depression symptoms in Polish youth. An attempt was made to assess the significance of external stressors and internal potential resources for maintaining psychological balance among young people.

**Methods:**

The study was conducted using the Revised Children’s Anxiety and Depression Scale (RCADS), the Questionnaire of Intrapersonal, Interpersonal, and World Attitudes (QIIWA), as well as a survey created by the researchers containing questions about well-being in relation to the pandemic, remote learning, and the ongoing war in Poland’s neighboring country. Ethical approval for the planned research was obtained from the Bioethics Committee. The study included 945 adolescents aged 11–15 years (*M* = 13.10; SD = 1.11), representing a sample of youth from 14 regions of Poland.

**Results:**

The results and analyses are presented from three perspectives: (A) the occurrence of anxiety and depression symptoms in relation to the gender and age of the respondents, (B) the significance of situational experiences, such as the COVID-19 pandemic (Q1), remote learning (Q2), and the outbreak of the war in Ukraine (Q3) for the occurrence of anxiety and depression symptoms in Polish youth, and (C) other determinants of anxiety and depression symptoms in Polish youth, such as intrapsychic variables including self-esteem, satisfaction with relationships with others, and views on the world and life.

**Discussion:**

It can be concluded that in the case of young people, maintaining mental health under the influence of social stressors such as the pandemic and the ongoing war in Ukraine depends mainly on intrapsychic variables, including the beliefs and attitudes of young people towards themselves, toward other people, the world and life.

## Introduction

The COVID-19 pandemic has significantly impacted mental health, leading to an increase in depression, anxiety, and other mental health issues. Numerous studies have shown that the occurrence of mental disorders among children and adolescents has increased both during and after the COVID-19 pandemic. This increase can be seen globally (1–4) and specifically in Poland (5–7). According to WHO data (1), approximately 15–20% of adolescents have recently been identified with different mental health problems. A global meta-analysis conducted by Racine et al. (2) revealed that in the first year of the COVID-19 pandemic, about 1 in 4 young people worldwide experienced symptoms of clinical depression, and 1 in 5 experienced anxiety symptoms. These figures were found to be twice as high compared to the pre-pandemic levels. Additionally, a UNICEF analysis (3) on the mental health situation of children and adolescents in Europe and factors which influence their well-being indicated that 10.8% of individuals aged 10–19 years (approximately 9 million teenagers in Europe) were diagnosed with mental disorders. According to UNICEF’s analysis, the prevalence of mental disorders among children and adolescents in Poland is comparable to European data, which suggests that over 409,000 teenagers (181,000 girls and 228,000 boys) suffer from mental health issues. The UNICEF report (3) on the condition of young people during the pandemic also highlighted several negative consequences of this phenomenon: 27% of teens reported anxiety, and 15% depression. UNICEF (3) estimated that in 2021, a total of 630,000 children in Poland required specialist psychological and psychiatric assistance, which was also confirmed by other reports (5).

A systematic review of studies regarding the impact of the pandemic on adolescent’s mental health revealed high rates of depression and anxiety among the youth due to social isolation, suspension of regular school and extracurricular activities, and pandemic restrictions. This led to the youth’s reduced participation in various life situations (4, 8–11). Additionally, in February 2022, while the COVID-19 pandemic was still ongoing, the war in Ukraine began (12). In Poland, this caused a potential security threat due to the proximity to Ukraine and exposed Poles to some traumatic experiences resulting from the war through direct contact with a significant number of war refugees (13). The prolonged and unpredictable nature of these non-normative events significantly weakened the mental condition of children and adolescents (14–16).

### Psychosocial determinants of emotional discomfort in adolescents

Aside from highlighting that global social stressors, such as pandemics and armed conflicts, have negative effects on the mental health of young people, literature also seeks factors that may help mitigate these psychological impacts. Studies on the effects of COVID-19 and the Russo-Ukrainian war on stress and anxiety in students indicate that both the pandemic and the war are sources of stress and anxiety, emphasizing the significance of gender as a primary risk factor (17). Additionally, Lass-Hennemann et al. (18) have investigated the impact of COVID-19 and Russian armed aggression on Ukraine on the mental health of the Finnish population. Their findings underscored the significance of anxiety and psychological stress in response to these global stressors and highlighted their adverse effects on mental health (18). Similar results had been found by Zhang et al. (19), which emphasized the impact of global crises, such as climate change, the COVID-19 pandemic, and the Russo-Ukrainian war, on the mental health of youth in Germany.

Specific studies in Poland on the pandemic’s impact on adolescent mental health showed that Polish teenagers experienced higher levels of anxiety and depression compared to their Chinese counterparts (4). This research indicates that young people are particularly vulnerable to stressors of such a global and vast nature, raising concerns about their future development. The increasing number of mental health problems in this age group suggests the need for a better understanding of the threats to adolescent mental health and the determinants and factors enabling the improvement of their mental well-being (20). This raises questions about which risk factors particularly contribute to negative mental health effects among youth in Poland. Are they events that disrupt social security, such as the COVID-19 pandemic and the ongoing war in Ukraine? Why do young people, compared to other age groups, struggle significantly to adapt to new, stressful environmental conditions?

One possible answer to these questions is that adolescence is a time of turbulent changes, both external and intrapsychic, in response to new social challenges. The developmental crisis of this life stage, characterized by reduced psychological balance due to developmental changes, can later manifest into having difficulties in functioning amid additional stressors (21, 22). Given the complexity of adolescence, the balance of burdens and protective factors, along with individual susceptibility and resilience, determines the individual’s mental well-being and strives to have sufficient balance to accomplish subsequent developmental tasks (23, 24). Attitudes toward oneself, others, and the world can be both sources of reinforcement and facilitators of social support utilization, or causes of passivity, isolation, and social distance, ultimately weakening the psychological resources of youth (25). Adequate self-esteem, a sense of security and social support, as well as a prosocial attitude, and a sense of self-efficacy and agency, are considered fundamental properties associated with resilience during development. These factors enable young individuals to believe in themselves, trust themselves, rely on the help and kindness of others, and maintain hope that their goals and aspirations are achievable despite obstacles (23, 26).

They are internal conditions which depend on the youth’s mental well-being. This is defined as the consciousness of an individual regarding their own potential, ability to make an effort to overcome challenges they may encounter, being productive in their personal and social life as well as trying use their skills to have an impact on society (27, 28). Maintaining subjective well-being in adolescents is essential for their psychological balance (29). Negative attitudes and a sense of deficiency of personal attractiveness, agency, and limited access to support can hinder maintaining balance in response to external stressors (26).

The exceptionally dynamic changes in adolescence regarding identity formation, increasing individualization, the intense transformation of knowledge about oneself, others, and the world, and the changing nature of relationships require consideration of many aspects (30). For a comprehensive assessment of mental health, it is essential to evaluate both distress and mental well-being, as confirmed in an Australian study of a significant group of youth aged 15–25 years (31). The salutogenic approach indicates that maintaining good mental health is possible when individuals possess sufficient competencies and resource potential to effectively cope with various life situations (29, 32, 33). This claim is supported by studies of a large group of Polish youth during the pandemic, where, despite most adolescents reporting feeling mentally worse after school closures, a group of students (about 17%) indicated better well-being under these circumstances (34). Similar findings were observed in other Polish studies during this period (5). This suggests that some youth found reinforcing aspects in relationships, such as family bonds or a sense of being, needed, compared to their peers (6, 35). Additionally, paradoxically, the pandemic provided better learning conditions for some, particularly those who struggle with in-person classes. During online learning, they appreciated the greater ability to focus, complete tasks at their own pace, and increased comfort without the need for waking up early, school noise, and other additional time burdens (36). This perspective highlights the role of individual adaptive abilities, which in the overall difficult experience, allowed youth to limit resource losses in the mental health domain. Thus, diverse experiences in challenging situations may result in negative consequences for some individuals without adverse effects for others (37, 38).

In response to the complex and specific external challenges influencing the functioning of contemporary youth in Poland, a comprehensive research study has been initiated. The objective of this study was to determine whether general social stressors, such as the experience of the COVID-19 pandemic and the war in Ukraine, as well as intrapsychic factors in the form of beliefs about oneself, others, life, and the world, are significant for the occurrence of anxiety and depression symptoms among Polish youth. To address this, the following research questions were formulated:

1. Is the occurrence of anxiety and depression symptoms among Polish youth related to situational variables, namely the experience of the pandemic and the war in Ukraine?
2. Are situational variables, specifically the experience of the pandemic and the war in Ukraine, causes of the occurrence of anxiety and depression symptoms among the youth studied?
3. Is the occurrence of anxiety and depression symptoms among Polish youth associated with intrapsychic variables, such as self-esteem, relationship satisfaction with others, a sense of self-efficacy, and the perception of the meaningfulness of the world?
4. Can the occurrence of anxiety and depression symptoms among Polish youth be predicted based on selected intrapsychic variables, including attitudes toward oneself, others, life, and the world?
5. Do selected intrapsychic variables, such as attitudes toward oneself, others, life, and the world, play a differentiating role in the occurrence of anxiety and depression symptoms among Polish youth?
6. Is the occurrence of anxiety and depression symptoms among Polish youth associated with age and gender within the studied group?

The approach undertaken to answer the formulated research questions is presented below.

## Materials and methods

### Research group

The study was conducted in May and June 2022 on a representative sample of Polish youth across 14 out of 16 voivodeships in Poland. A total of 945 adolescents participated in the study, including 520 girls (55.03%) and 425 boys (44.97%). The participants ranged in age from 11 to 15 years (*M* = 13.10; SD = 1.11), with the sample comprising of 69 11-year-olds (7.30%), 230 12-year-olds (24.34%), 295 13-year-olds (31.22%), 243 14-year-olds (25.71%), and 108 15-year-olds (11.43%). The sampled population was randomly selected from primary schools in rural areas (34.39%), small towns with up to 20,000 inhabitants (21.06%), large cities with over 20,000 inhabitants (24.13%), and metropolitan areas with over 100,000 inhabitants (20.42%). Additionally, the number of participants in each age group varied according to their parents’ education level (primary, vocational, secondary, higher).

The study was conducted in groups, within school classrooms. The questionnaires were prepared in electronic form, and each participating student received an individual identification code (ID) to access the online form. The survey included all necessary instructions, and trained personnel (school counselors or psychologists) were present to provide additional guidance. Parental or legal guardian consent was obtained for each student’s participation, and the students themselves also gave informed consent.

### Research tools

The following research instruments were used in the study:

1. **The Revised Child Anxiety and Depression Scale (RCADS)** by Chorpita et al. (38), translated into Polish by Skoczeń et al. (39). This tool aims to assess the severity of anxiety and depressive symptoms in children, providing a global anxiety and depression score (GLOBAL) and specific indices for Separation Anxiety Disorder (SAD), Generalized Anxiety Disorder (GAD), Panic Disorder (PD), Social Phobia (SOC), Obsessive-Compulsive Disorder (OCD), and Major Depressive Disorder (MDD) based on DSM-5 criteria (39). The original English version is designed for children and adolescents aged 3–17.5 years. The Polish version was tested on a sample of 501 children and adolescents aged 8–14 years (39). The questionnaire consists of 47 statements which require the respondent to indicate the frequency of each symptom on a scale from “Never” to “Always.” The reliability of the questionnaire, assessed by the authors using Bagozzi’s formula, is 0.96, while in this study, it was measured using Cronbach’s alpha coefficient, yielding a value of 0.99.
2. **The Questionnaire of Intrapersonal, Interpersonal, and World Attitudes (QIIWA)** by Aksman and Wysocka (24). This tool measures intrapersonal attitudes (self-image and general self-esteem), interpersonal attitudes (the image of others and relationships with them), and attitudes toward the world and one’s own life. Intrapersonal attitudes encompass beliefs about oneself, while interpersonal attitudes relate to beliefs about functioning in relationships and attitudes toward others. Attitudes toward the world reflect beliefs about the world’s meaningfulness and benevolence, and attitudes toward one’s life reflect beliefs about self-efficacy and control versus learned helplessness. The questionnaire is designed for youth aged 11–16 years, based on cognitive personality theory, which emphasizes how individuals understand themselves and the world and act accordingly. The questionnaire contains 60 statements rated on a scale from “Strongly Disagree” (1 point) to “Strongly Agree” (4 points). The reliability of the scale is satisfactory, with a Cronbach’s alpha of 0.87, and in this study, it was 0.99.
3. **Self-prepared Survey** – it examined reactions to events related to the pandemic and the war in Ukraine, rated on a scale from 0 (no impact) to 10 (very strong impact).

- Question 1: How much did the COVID-19 pandemic situation impact you?
- Question 2: How would you rate the stress you usually feel during online learning?
- Question 3: How much did the situation related to the war in Ukraine impact you?

### Statistical analysis

The statistical analysis was performed using the STATISTICA software package. The following methods were employed: Kruskal-Wallis ANOVA (a non-parametric test used for comparing multiple groups of different sizes), Student’s *t*-test for group differences (a parametric test used to compare two groups of similar sizes), Pearson’s correlation coefficient (for examining the relationship between quantitative variables with a normal distribution and linear dependency), multiple regression analysis (used for predicting the value of a dependent variable based on several independent variables), and k-means clustering (for finding independent clusters of variables).

## Results

The results of the conducted research will be presented in accordance with the chronology derived from the content of the research questions.

### The importance of situational variables for anxiety and depression symptoms in polish youth

The presence of anxiety and depression symptoms in the studied group of adolescents was largely positively associated with experiences related to the COVID-19 pandemic (*r* = 0.261; *p* < 0.001), online learning (*r* = 0.432; *p* < 0.001), and the outbreak of the war in Ukraine (*r* = 0.232; *p* < 0.001). The strength of these associations ranged from weak to moderate, with a general trend showing that greater concern about the COVID-19 pandemic, online learning, and the war in Ukraine corresponded to a higher incidence of anxiety and depression symptoms among the respondents (Table 1).

**Table 1:** Correlations between concern about social situations and the occurrence of anxiety and depression symptoms in the studied adolescents (*N* = 945).

Variables		Q1	Q2	Q3
RCADS	SOC	0.190***	0.338***	0.183***
	PD	0.223***	0.324***	0.187***
	MDD	0.286***	0.379***	0.291***
	SAD	0.089**	0.292***	0.088**
	GAD	0.232***	0.325***	0.193***
	OCD	0.237***	0.388***	0.212***
	GLOBAL	0.261***	0.432***	0.232***

^*^p < 0.05; ^**^p < 0.01; ^***^p < 0.001.

SOC, Social Phobia; PD, Panic Disorder; MDD, Major Depressive Disorder; SAD, Separation Anxiety; GAD, Generalized Anxiety; OCD, Obsessive-Compulsive Disorder; GLOBAL, the total anxiety and depression index; L, Scale of Lying; N, Neuroticism Scale; Q1, anxiety about the pandemic; Q2, remote learning; Q3, the outbreak of war in Ukraine.

Furthermore, the concern felt by the surveyed youth, particularly related to the COVID-19 pandemic and online education, considering the concurrent experience of the outbreak of the war in Ukraine, was a significant predictor of discomfort associated with anxiety and depression. Situational variables explained approximately 19.6% of the variance in discomfort in the studied group (Table 2).

**Table 2:** Emotional discomfort expressed by the global RCADS score in the group of adolescents (*N* = 945) regarding concern about the COVID-19 pandemic, online education, and the outbreak of the war in Ukraine.

Predictors	b*	Std. error of b*	*b*	Std. error of *b*	*t*(894)	*p*-value
Constant term			26.06	1.50	17.41	<0.001
Q1	0.08	0.04	0.75	0.37	2.02	0.044
Q2	0.38	0.03	3.73	0.32	11.72	<0.001
Q3	0.05	0.04	0.43	0.33	1.28	0.202

*F*(3, 894) = 73.87; *p* < 0.001; Std. error of estimation: 23.47; *R*^2^ = 0.196.

Q1, anxiety about the pandemic; Q2, remote learning; Q3, the outbreak of war in Ukraine.

### The importance of intrapsychic variables for anxiety and depression symptoms in polish youth

Symptoms related to anxiety and depression were examined to see if they are interwoven with intrapsychic variables such as self-esteem, satisfaction with relationships, and worldview in the Polish youth. In the studied group of adolescents, there were significant negative correlations between intrapsychic and interpersonal attitudes—attitudes toward oneself (*r* = −0.226; *p* < 0.001), others (*r* = −0.423; *p* < 0.001), the world (*r* = −0.417; *p* < 0.001), and one’s life (*r* = −0.563; *p* < 0.001)—and the severity of anxiety and depression symptoms. This indicates that lower self-esteem, less satisfaction with interpersonal relationships, a lower sense of meaningfulness and benevolence of the world, and a lower sense of self-efficacy were associated with more anxiety and depression symptoms. These correlations ranged from weak to moderate (Table 3).

**Table 3:** Correlations between attitudes toward oneself, others, the world, and life and the occurrence of anxiety and depression symptoms in the studied adolescents (Pearson’s r; *N* = 945).

QIIWA					
Variables		S	IB	BW	BL
RCADS	SOC	−0.192***	−0.388***	−0.416***	−0.483***
	PD	−0.078***	−0.189***	−0.155***	−0.317***
	MDD	−0.111***	−0.261***	−0.319***	−0.422***
	SAD	−0.222***	−0.392***	−0.382***	−0.498***
	GAD	−0.136***	−0.253***	−0.261***	−0.382***
	OCD	−0.166***	−0.299***	−0.280***	−0.466***
	GLOBAL	−0.226***	−0.423***	−0.417***	−0.563***

^*^p < 0.05; ^**^p < 0.01; ^***^p < 0.001.

SOC, Social Phobia; PD, Panic Disorder; MDD, Major Depressive Disorder; SAD, Separation Anxiety; GAD, Generalized Anxiety; OCD, Obsessive-Compulsive Disorder; GLOBAL, the total anxiety and depression index; S, self-esteem; IB, interpersonal beliefs; BW, beliefs about the world; BL, beliefs about life.

It was also investigated whether intrapsychic variables, namely self-esteem, satisfaction with relationships, and worldview, could predict the occurrence of anxiety and depression symptoms. The configuration of these attitudes was a significant predictor of the intensity of emotional discomfort among the respondents. The independent variables explained nearly 36% of the variance in the overall severity of anxiety and depression symptoms in the studied group (Table 4).

**Table 4:** Emotional discomfort expressed by the global RCADS score in the group of adolescents (*N* = 945) concerning the configuration of individual variables.

Predictors	b*	Std. error of b*	*b*	Std. error of *b*	*t*(894)	*p*-value
Constant term			130.22	4.98	26.16	<0.001
S	0.18	0.04	0.46	0.09	5.02	<0.001
IB	−0.16	0.04	−0.47	0.12	−4.04	<0.001
BW	−0.14	0.04	−0.76	0.20	−3.81	<0.001
BL	−0.49	0.04	−2.53	0.19	−13.21	<0.001

*F*(4, 830) = 116.20; *p* < 0.001; Std. error of estimation: 20.74; *R*^2^ = 0.356.

S, self-esteem; IB, interpersonal beliefs; BW, beliefs about the world; BL, beliefs about life.

### The differentiating role of selected attitudes and beliefs regarding emotional discomfort related to anxiety and depression in polish youth

**Figure 1:**
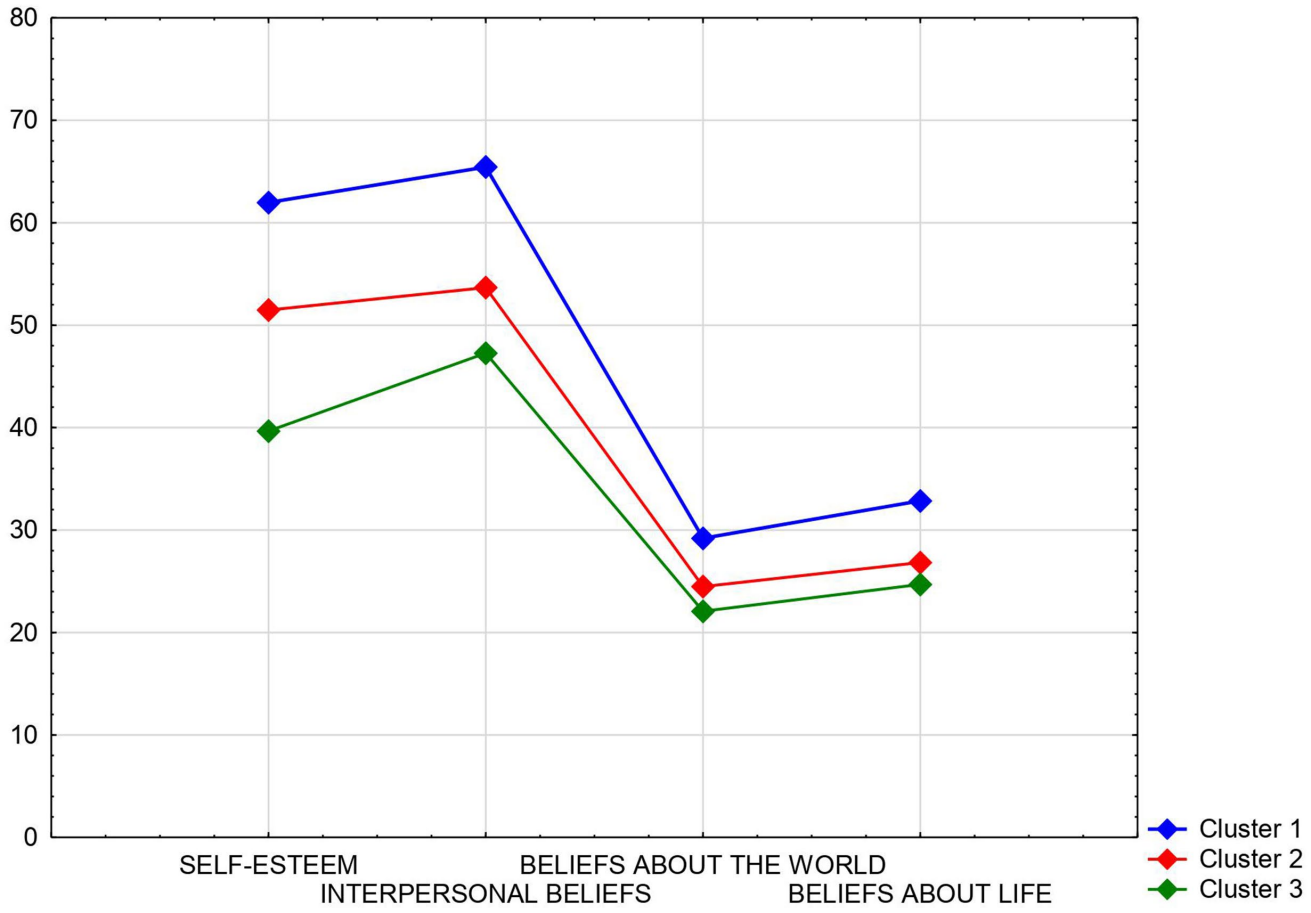
Graph showing differences between the means in different clusters based on the respondents’ attitudes toward themselves, others, life, and the world, focusing on the overall intensity of emotional discomfort related to anxiety and depression.

It was also examined whether the individual beliefs of the surveyed youth regarding themselves, others, life, and the world played a differentiating role concerning their experienced emotional discomfort. For this purpose, k-means clustering analysis was used, and three clusters of respondents were identified based on the description of their attitudes and beliefs (Figure 1).

Youth in cluster 1 were characterized by significantly higher self-esteem, higher levels of satisfaction due to support from others, more prosocial attitudes, and significantly higher self-efficacy and lack of helplessness compared to respondents in clusters 2 and 3. Cluster 3 included individuals with the lowest self-esteem, lower satisfaction with relationships, a significantly lower sense of efficacy in life, and significantly lower perceived meaningfulness and comprehensibility of the world compared to individuals in the other two clusters (Table 5).

**Table 5:** The significance of the differences between the means of the three clusters considering attitudes toward oneself, others, the world, and life in the studied adolescents.

Variables	Cluster 1 (*n* = 180)		Cluster 2 (*n* = 385)		Cluster 3 (*n* = 270)		*H* (2, *N* = 835)	*p*-value
	*M*	SD	*M*	SD	*M*	SD		
**QIIWA**								
S	61.98 ab	6.39	51.48 ac	4.83	39.66 bc	5.70	607.90	<0.001
IB	65.45 ab	5.92	53.67 ac	5.33	47.28 bc	6.28	461.96	<0.001
BW	29.21 ab	4.59	24.48 ac	3.45	22.07 bc	4.25	223.50	<0.001
BL	32.85 ab	3.72	26.84 ac	3.74	24.70 bc	4.45	297.56	<0.001

S, self-esteem; IB, interpersonal beliefs; BW, beliefs about the world; BL, beliefs about life.

The identified clusters were examined to decide whether the differentiated characteristics of the respondents’ beliefs were related to the severity of anxiety and depression symptoms and the overall emotional discomfort experienced. Youth with more favorable beliefs experienced significantly lower severity of anxiety and depression symptoms and overall emotional discomfort compared to adolescents with lower self-esteem, less satisfaction with relationships, lower self-efficacy, and beliefs about the lower meaningfulness and friendliness of the world (Table 6).

**Table 6:** The significance of the differences between the means in terms of the occurrence of anxiety and depression symptoms and overall discomfort expressed by the global score in clusters identified based on respondents’ attitudes toward themselves, others, the world, and life.

Variables	Cluster 1 (*n* = 180)		Cluster 2 (*n* = 385)		Cluster 3 (*n* = 270)		*H* (2, *N* = 941)	*p*-value
	*M*	SD	*M*	SD	*M*	SD		
**RCADS**								
SOC	2.51 ab	1.60	3.51 ac	1.86	3.96 bc	2.26	60.89	<0.001
PD	1.13 ab	1.17	1.76 a	1.60	1.80 b	1.67	24.73	<0.001
MDD	5.15 ab	2.91	6.66 a	3.26	6.73 b	3.79	31.38	<0.001
SAD	1.00 ab	1.36	2.14 ac	1.87	2.55 bc	2.25	79.91	<0.001
GAD	1.58 ab	1.57	2.34 a	1.83	2.51 b	2.18	27.36	<0.001
OCD	1.46 ab	1.37	2.36 ac	1.91	2.74 bc	2.38	38.51	<0.001
GLOBAL	24.78 ab	16.42	41.15 ac	23.18	45.78 bc	26.42	86.70	<0.001

SOC, Social Phobia; PD, Panic Disorder; MDD, Major Depressive Disorder; SAD, Separation Anxiety; GAD, Generalized Anxiety; OCD, Obsessive-Compulsive Disorder; GLOBAL, the total anxiety and depression index.

It was proven then that the adolescents’ beliefs toward themselves, others, life, and the world have a significant impact on their self-esteem in the emotional sphere.

### The occurrence of anxiety and depression symptoms depending on the age and gender of the respondents

Subsequently, the role of age and gender in the occurrence of anxiety and depression in the studied youth was examined. Previous studies indicated that age and gender are significant factors in the emotional development of adolescents. Therefore, an analysis of differences between groups based on these variables was conducted.

Significant age-related differences in the occurrence of anxiety and depression symptoms in the studied youth were observed only in separation anxiety disorder (SAD), which was significantly less severe in the group of 11-year-olds compared to the group of 15-year-olds [*H* (4, *N* = 945) = 11.90; *p* = 0.018] (Table 7).

**Table 7:** Characteristics of the studied group (*N* = 945) regarding the occurrence of anxiety and depression symptoms separated by age.

	11		12		13		14		15		*H* (4, *N* = 945)	*p*-value
	*M*	SD	*M*	SD	*M*	SD	*M*	SD	*M*	SD		
**RCADS**												
SOC	2.91	2.01	3.37	1.96	3.49	2.04	3.70	2.09	3.73	2.18	8.30	0.081
PD	1.67	1.75	1.66	1.53	1.56	1.51	1.66	1.51	1.89	1.86	2.11	0.715
MDD	5.54	3.68	6.53	3.42	6.64	3.43	6.43	3.47	5.95	3.51	7.77	0.100
SAD	1.57a.b	1.99	1.90	1.88	2.11	2.03	2.28a	2.08	2.30b	2.17	11.90	0.018
GAD	2.09	2.20	2.17	1.88	2.24	1.91	2.33	1.90	2.48	2.28	2.90	0.575
OCD	2.03	2.16	2.42	2.04	2.38	2.07	2.28	2.01	2.39	2.30	3.75	0.441
GLOBAL	33.30	27.23	39.89	25.09	39.93	25.92	41.28	26.54	40.91	29.44	6.69	0.153

SOC, Social Phobia; PD, Panic Disorder; MDD, Major Depressive Disorder; SAD, Separation Anxiety; GAD, Generalized Anxiety; OCD, Obsessive-Compulsive Disorder; GLOBAL, the total anxiety and depression index.

Among the surveyed adolescents, girls exhibited higher severity of anxiety and depression symptoms, although the differences were weak to moderate (Table 8).

**Table 8:** Characteristics of the studied group (*N* = 945) regarding the occurrence of anxiety and depression symptoms separated by gender.

	Boys		Girls		*t*(943)	*p*-value	Cohen’s *d*
	*M*	SD	*M*	SD			
**RCADS**							
SOC	2.93	2.00	3.97	1.98	−7.963	<0.001	0.52
PD	1.37	1.57	1.89	1.54	−5.100	<0.001	0.33
MDD	5.27	3.30	7.33	3.34	−9.463	<0.001	0.62
SAD	1.59	1.84	2.49	2.08	−6.905	<0.001	0.46
GAD	1.94	1.98	2.52	1.92	−4.568	<0.001	0.30
OCD	1.89	1.95	2.71	2.11	−6.178	<0.001	0.40
GLOBAL	32.25	24.87	46.14	26.02	−8.328	<0.001	0.55

SOC, Social Phobia; PD, Panic Disorder; MDD, Major Depressive Disorder; SAD, Separation Anxiety; GAD, Generalized Anxiety; OCD, Obsessive-Compulsive Disorder; GLOBAL, the total anxiety and depression index.

### The intensity of situational stress depending on the age and gender of the studied youth

It was also examined whether age and gender impacted the intensity of situational stress among the surveyed adolescents based on an analysis of group differences. It was found that the level of concern related to the COVID-19 pandemic, online learning, and the outbreak of the war in Ukraine was similar across age subgroups between 11 and 15 years old (Table 9).

**Table 9:** Characteristics of the studied group (*N* = 945) regarding concerns about social situations around the world separated by age of the respondents.

	11		12		13		14		15		*H* (4, *N* = 898)	*p*-value
	*M*	SD	*M*	SD	*M*	SD	*M*	SD	*M*	SD		
**Survey**												
Q1	3.39	2.69	3.51	2.74	3.76	2.82	3.32	2.80	3.35	2.98	3.97	0.410
Q2	2.39	2.82	2.46	2.44	2.40	2.60	2.75	2.94	2.35	2.82	2.34	0.674
Q3	4.44	3.22	4.63	2.98	5.01	3.00	4.67	3.11	4.61	3.16	3.01	0.556

Q1, anxiety about the pandemic; Q2, remote learning; Q3, the outbreak of war in Ukraine.

Girls revealed a higher concern about the COVID-19 pandemic (*t* = −5.180; *df* = 943; *p* < 0.001) and the outbreak of the war in Ukraine (*t* = −6.815; *df* = 943; *p* < 0.001), as well as significantly higher stress related to remote learning (*t* = −7.939; *df* = 943; *p* < 0.001). The strength of these effects was moderate (Table 10).

**Table 10:** Characteristics of the studied group (*N* = 945) regarding concerns about social situations separated by gender of the respondents.

	Boys		Girls		*t*(943)	*p*-value	Cohen’s *d*
	*M*	SD	*M*	SD			
**Survey**							
Q1	2.99	2.87	3.95	2.67	−5.180	<0.001	0.35
Q2	1.73	2.44	3.12	2.74	−7.939	<0.001	0.54
Q3	3.99	3.15	5.36	2.84	−6.815	<0.001	0.46

Q1, anxiety about the pandemic; Q2, remote learning; Q3, the outbreak of war in Ukraine.

### Characteristics of attitudes toward oneself, others, life, and the world concerning age and gender of the studied adolescents

In the next step, the significance of age and gender on the characteristics of the surveyed youth’s attitudes in various aspects of functioning was analyzed. Attitudes toward oneself, understood as self-esteem, attitudes toward others, meaning prosocial attitudes and a sense of reciprocity, and attitudes toward one’s own life, understood as self-efficacy and lack of helplessness, were similar across the adolescent’s age subgroups. Only attitudes toward the world, understood as beliefs about its meaningfulness and friendliness, were highest in the youngest group and lowest in the oldest groups, i.e., 14-year-olds and 15-year-olds [*H* (4, *N* = 898) = 12.68; *p* = 0.013] (Table 11).

**Table 11:** Characteristics of the studied group (*N* = 945) regarding attitudes toward oneself, others, the world, and life separated by age of the respondents.

	11		12		13		14		15		*H* (4, *N* = 898)	*p*-value
	*M*	SD	*M*	SD	*M*	SD	*M*	SD	*M*	SD		
**QIIWA**												
S	50.58	12.76	48.66	10.26	50.61	9.13	49.99	9.45	50.09	9.80	5.39	0.250
IB	55.65	9.13	54.18	8.65	54.80	8.19	53.26	9.49	53.41	8.14	6.67	0.154
BW	26.54a.b	4.92	25.17	4.76	24.76	4.57	24.16a	4.76	23.96b	4.73	12.68	0.013
BL	27.56	5.33	27.53	4.55	27.56	5.05	27.29	5.27	27.20	4.76	0.77	0.942

S, self-esteem; IB, interpersonal beliefs; BW, beliefs about the world; BL, beliefs about life.

Overall self-esteem was significantly higher in the group of surveyed girls than in the boys (*t* = −2.596; *df* = 834; *p* = 0.010), but their attitudes toward the world—i.e., a sense of meaningfulness and friendliness of the environment (*t* = 2.055; *df* = 834; *p* = 0.040)—and attitudes toward life—i.e., self-efficacy and lack of helplessness (*t* = 3.014; *df* = 834; *p* = 0.003)—were significantly lower compared to these attitudes in the surveyed boys (Table 12).

**Table 12:** Characteristics of the studied group (*N* = 945) regarding attitudes toward oneself, others, the world, and life separated by gender of the respondents.

	Boys		Girls		*t*(834)	*p*-value	Cohen’s *d*
	*M*	SD	*M*	SD			
**QIIWA**							
S	48.96	10.07	50.73	9.56	−2.596	0.010	0.18
IB	54.03	8.09	54.22	9.24	−0.302	0.762	–
BW	25.08	4.72	24.41	4.74	2.055	0.040	0.14
BL	28.00	4.68	26.97	5.17	3.014	0.003	0.21

S, self-esteem; IB, interpersonal beliefs; BW, beliefs about the world; BL, beliefs about life.

## Discussion

The presented research project aimed at analyzing the role of situational and intrapsychic variables on the emotional state of Polish adolescents aged 11–15. Situational variables include broad social stressors such as the COVID-19 pandemic and its consequences, like remote learning, as well as the ongoing war in Ukraine. Intrapsychic variables considered in the analysis encompass adolescents’ attitudes toward themselves, others, and life in general. The emotional state of the studied youth in Poland was described as an occurrence of symptoms of anxiety and depressive disorders which were perceived as discomfort. It is known that the emotional balance of a young person is influenced by multiple factors (22, 40). It may be related to the nature of experiences and the individual’s psychological resilience to a traumatizing impact of some difficult events, and it varies by age and gender.

Summarizing the key findings of the conducted study (noting that these findings are discussed in greater detail in the subsequent sections), it is important to highlight that some significant social stressors, such as the COVID-19 pandemic, including remote learning and the war in Ukraine, were strongly associated with increased symptoms of anxiety and depression among Polish adolescents. These circumstances accounted for 19.6% of the variance in the observed distress levels among the participants. At the same time, intrapsychic characteristics, such as self-esteem, positive attitudes toward others, the world, and life, proved to be significantly more impactful, showing a strong negative correlation with the prevalence of anxiety and depression symptoms. These factors explained as much as 36% of the variance in overall symptom severity within the studied group. The findings suggest that adolescents’ ability to cope with challenging situations is closely tied to beliefs and perceptions about themselves, others, and reality. The importance of these intrapsychic characteristics is nearly twice as high as that of the aforementioned external factors. More adaptive beliefs and attitudes represent a substantial resource within the studied adolescent population.

The pandemic threatened human being’s physical well-being and significantly altered teenagers’ lifestyles. Prolonged school closures led to social isolation, and the shift to remote learning increased psychological stress, which most young people highlighted as a particularly traumatizing element of this experience (6, 41). Limited opportunities for social contact and the loss of a daily routine negatively impacted the youth’s mental health (42). According to UNESCO (43) data, almost half of the world’s adolescent population faced additional sources of oppression, such as the death of close family members, financial instability in households, and lingering uncertainty and anxiety about the future (32, 44, 45).

Specific to the Polish youth population was the simultaneous experience of the war in their neighboring country along with the pandemic. These co-occurring non-normative and security-threatening situations created circumstances so nerve-racking that they became a predictor of the variability of anxiety and depressive symptoms. This indicates the significant negative impact of the combination of these external stressors on the mental health of adolescents in Poland (40, 46, 47).

Among the resources shaping an individual’s resilience to stressful events and difficult circumstances, self-efficacy and action effectiveness are distinguished. They are perceived as an effect of personal experiences which shape life attitudes (48). They are crucial for maintaining good health, as stronger self-efficacy correlates with a greater tendency to effectively cope with various stressful situations to achieve one’s goals (49). The ability to adapt to changes under stress is an indicator allowing for predicting resilience (50).

The findings of the conducted study align with analyses reported in other countries (with reference to related research). For instance, a systematic review of 16 quantitative studies conducted between 2019 and 2021, involving 40,076 participants from various countries, revealed that adolescents from diverse backgrounds experienced elevated levels of anxiety, depression, and stress associated with the COVID-19 pandemic (48). This review also highlighted that social support, good coping skills, home quarantine, and positive parent–child relationships had a beneficial impact on the adolescents’ mental health during this period of crisis. Moreover, meta-analyses conducted by Racine and colleagues (2) indicated that one in four young people worldwide experienced clinically elevated symptoms of depression during the first year of the COVID-19 pandemic, while one in five exhibited clinically elevated symptoms of anxiety in the same time frame. These estimates, which increased over time, were twice as high compared to pre-pandemic levels (2).

The significance of the regional, and even global crisis, related to the armed conflict in Ukraine was also noted by researchers of the German youth population, who indicated this factor as more strongly associated with anxiety, compared to the effects of the pandemic and climate change (18). Even in the more faraway Netherlands, information about the ongoing war in Ukraine available in the media was a prominent source of threat and increased anxiety among the surveyed adolescents. Additionally, access to information about military actions was positively associated with stress intensity (51).

Similarly, studies conducted in Finland (17) identified high levels of stress and anxiety among young people related to both the pandemic and the war. In contrast, China, where the pandemic was also a significant stressor, reported lower levels of anxiety and depression compared to Poland. This disparity may stem from cultural differences and variations in mental health support systems (4). These comparisons provide valuable insights into the unique experiences of Polish adolescents while also situating the findings of this research within the broader global context of the pandemic.

The experience of the ongoing war, potential, and prolonged threats, even if not directly affecting the youth, can threaten their well-being (20). They contradict the basic developmental needs of the adolescents and their right to grow up in a safe and predictable environment. Psychologically weaker youth, who experiences less support and believes in their lower resilience, may be more susceptible to disorders due to unfavorable external conditions (24, 36). The study showed that youth discomfort could manifest in various ways, including generalized anxiety disorder (GAD), panic disorder (PD), separation anxiety disorder (SAD), social anxiety disorder (SOD), obsessive-compulsive disorder (OCD), or depression symptoms (MDD). Thus, the traumatic effects of the mentioned situations appear non-specific and affect young people, compromising their resilience depending on their individual predispositions (25).

Previous researchers’ observations about the variability of psychological well-being concerning adolescents’ gender were confirmed in the studied group. Girls reported more symptoms of anxiety and more concern about the social crisis related to the COVID-19 pandemic and its consequential online learning and the outbreak of war in Ukraine. Similar results were obtained in earlier studies. The female gender was an element leading to a greater risk of developing anxiety and depression among Chinese youth during the pandemic (52, 53). Earlier reports from Poland also showed that negative indicators of the youth’s emotional condition more frequently concerned girls rather than boys (5, 6). Previous studies also indicate that adolescent girls, compared to boys, experience more intense emotions and greater emotional instability (54, 55). The difference in responses has biological roots due to neuroanatomical and functional differences in the amygdala, significant for emotional control (56). Additionally, girls are more inclined to admit experiencing unpleasant emotions and are even encouraged to do so, as per the cultural stereotypes and socialization process. In contrast, boys are generally mobilized to suppress emotional expression and self-control (57).

Less stability and resilience in girls to difficult experiences is also confirmed by their responses, which indicate greater concern about the COVID-19 pandemic, significantly higher stress from remote learning, and stronger concern about the war outbreak in Ukraine, in comparison to boys. All above mentioned events meet the criteria for a crisis situation (58). However, in the case of the war, its physical and psychological effects in Poland are more likely a result of indirect trauma exposure (59). Studies also show that the accumulation of stressors and their simultaneous occurrence, which particularly impacts the interpersonal context, significantly affects emotional well-being in a negative way, especially in girls (60).

Among these conditions, age and stage of adolescence are less significant. In the studied group, age only revealed its role in regard to the severity of separation anxiety symptoms. It significantly differentiates the youngest adolescents in the group, aged 11, who exhibit notably less severe symptoms compared to 15-year-olds. This result is surprising and contrary to expectations, as it is known that with age, young people generally improve their stress-coping abilities (22, 61). An explanation for this specific phenomenon is found among the results of this same study, specifically in the section indicating the diversity of beliefs of the studied youth regarding themselves, others, and the world and life.

It turns out that age-related differences are evident in the youth’s beliefs. These differences pertain to attitudes toward the world, specifically the belief in its meaningfulness and benevolence, which is the lowest among the oldest adolescents and significantly lower compared to the 11-year-olds. The belief that the world is organized sensibly and generally benevolent is an element of basic hope, according to Erikson (62). It is a manifestation of a positive resolution of the conflict between trust and mistrust in the world at the beginning of life, forming the basis for achieving subsequent developmental goals (62). The feeling of sensibility and comprehensibility of the world are also essential components of the sense of coherence, according to Antonovski (63), which means seeing the world as coherent and meaningful enough to safely engage in shaping one’s life within it. Trust in the world is limited among older adolescents compared to younger teenagers, which is possibly linked to greater doubts about whether they can meet the complex demands of the world and whether these demands are worth the effort. Less hope for fulfilling important needs may be associated with the intensification of the developmental crisis of transitioning from childhood to adulthood and the destabilized functioning of a young person before forming a new coherent self-image in the surrounding world (62, 64). In this context, the reluctance to distance themselves from parental care and the desire to remain in safe proximity to their parents may be more understandable, especially for 15-year-olds. Greater uncertainty may also result from increasingly complex cognitive assessments of reality, becoming more penetrating and realistic with age (20, 22). A complicated worldview may evoke a desire to withdraw under the protective wings of parents, especially since the coping maturity of 15-year-olds is still incomplete. The obtained results indicate fluctuations in the adolescents’ separation concerns while they become independent from their parents throughout adolescence. However, these concerns do not reach levels high enough to suggest a psychopathological intensity of anxiety. Seeking secure support in the parent–child relationship appears to reflect the utilization of resources available to the young individual rather than a sign of maladjustment (6, 35).

Therefore, the obtained results present a complex signal about the well-being of youth and their probable connections with beliefs, which may be conditioned by both the developmental stage and external situational factors.

The beliefs of the studied youth also vary by gender, allowing for a mapping of resources and areas requiring care for both girls and boys. Girls have a higher self-esteem index than boys, which is consistent with previous findings among the Polish youth (65). However, in other studies, self-esteem indicators regarding gender were opposite. For example, among 14-year-old adolescents in Bulgaria, girls had significantly more negative attitudes toward themselves compared to boys (66). In studies regarding Spanish youth, self-esteem varied by specific functioning aspects, with the overall self-esteem higher in girls but boys rating themselves higher in academic achievements, emotional stability, and parental relationships (67). Self-perception is crucial for good well-being and how emotions are experienced. The more unstable and unpleasant emotions, the lower the self-esteem (22, 25). Based on the obtained results, it appears to be an area of lesser resilience among the studied male youth.

Conversely, boys’ attitudes toward the world, considering resilience and emotional balance in difficult situations, are more favorable compared to girls. Similar divisions regarding the sense of coherence between girls and boys were also obtained in earlier studies (60). The world, in the beliefs and experiences of male youth, is more likely a source of pleasure and safety, and appears more understandable and coherent. This indicates the presence of basic hope, which makes it easier to overcome difficulties and crises in life. The level of faith in the world’s meaningfulness and general benevolence determines which coping strategies are used to handle potential disruptions in the established order of functioning. The stronger the hope, the more constructive the coping strategies will be (24, 62, 63).

According to the cognitive personality theory and classical cognitive theories, beliefs about one’s personality and the world play a crucial role in shaping one’s mental health (68). They form the basis for shaping one’s development path, predicting its functioning, and measuring individual differences and identity formation (24). These assumptions were confirmed in the conducted studies. It turned out that adolescents’ beliefs had greater predictive power regarding the occurrence of symptoms compared to situational variables. Attitudes toward oneself, others, the world and life explained nearly 36% of the variance in the overall intensity of anxiety and depression symptoms in the studied group. Therefore, the layout of belief content largely allows for predicting mental health, particularly concerning the emotional balance of youth.

A positive view of self, others, and the world is also characteristic of a resilient personality, which is a guarantee of resilience and coping efficiency in humans (69).

The differentiation in health prognosis among youth, depending on their belief characteristics, indicates this. The group of adolescents with the most positive attitudes toward themselves and others and with a sense of reciprocated kindness, world benevolence, and life effectiveness, experienced significantly fewer symptoms of anxiety, depression, and general discomfort compared to adolescents with less positive beliefs. This aligns with the researchers’ findings regarding dynamic coping with difficult situations, known as resilience. Resilient individuals exhibit higher self-esteem and self-efficacy, perceive the environment as benevolent, and view life as controllable and shapeable by the individual, who can be effective and influence its course (69, 70). They are also open toward others and capable of forming close and satisfying relationships (71). Resilience is also directly linked to more positive emotions and balanced affectivity (72). This image can be attributed to adolescents in this group, making it the only resilient one among the studied, as the beliefs of the remaining respondents, even those at the average group level and the more negative levels, were significantly less protective and associated with significantly higher emotional discomfort.

It is somewhat unexpected that concern about the pandemic and the outbreak of war emerged most strongly among adolescents with medium-positive attitudes compared to the whole group. Perhaps, at the root of these medium-intensity attitudes is an ambiguous assessment of oneself, others, the world, and life, making these individuals most susceptible to situational stress. Negative events, such as the pandemic and its consequential isolation and later the war outbreak, might confirm the anticipations of those with the most negative beliefs about threats and the negative aspects of life. If so, the negatively predisposed group does not experience increased discomfort because they consistently expect the world’s unkindness and life’s difficulties, and their negative vision seems confirmed in objectively difficult and burdensome circumstances of the pandemic and war (73). Their previous assumptions and the subjective view of reality remain consistent with the real experience of difficulties, therefore the strategies they used previously may be helpful when the situation becomes complicated. Of course, this can only work short-term, as general negative assumptions about oneself, others, the world, and life are significant predictors of anxiety and depression symptoms in young people. Simultaneously, the ambiguity and uncertainty of beliefs also emerge as risk factors for poorer mental health. According to the concept of coherence, a lower sense of comprehensibility, perceived coherence, and meaningfulness of functioning indicates a lower potential for coping with challenges, adversities, and burdens in life (62, 63).

## Limitations

One of the limitations of the presented research project is the fact that the study was conducted in a group setting using an online form while the students were at school. This means that the young participants answered the questions independently based on their own understanding, which might not have occurred in completely comfortable surroundings. Although the study was conducted in the presence of a researcher, it is likely that some respondents were content with their own assessment of the meaning and correctness of the questions, even if they did not fully understand the statement descriptions.

Another limitation is the questionnaire-based nature of the research. Relying on self-reporting provides information solely within the scope of the respondents’ self-awareness, with the accuracy being dependent on the participants’ level of insight. When it comes to questions about unpleasant experiences and psychological states, it should be assumed that the information might be incomplete. Additionally, no information was obtained from the guardians of the surveyed youth, which could have objectified the data obtained from the adolescents, representing another limitation of the presented results.

The study is representative only of young people who function normally and participate in primary school activities. It would be worthy to obtain similar knowledge about other age groups of children and adolescents, as well as among young people at increased risk of developing disorders. Given the dynamically changing social situation, it would be worthwhile to undertake longitudinal studies to observe the dynamics of young people’s adaptation/maladaptation to prolonged exposure to stress factors.

## Conclusion and implications

The results obtained in the presented studies conclude that an important task for fostering youth resilience is supporting adolescents in developing the most positive, yet realistic, self-concept consistent with their life conditions. Organizing the youth’s interpersonal world in such a way that allows for experiencing the benevolence of others, social kindness, and effectiveness in action is crucial. This positive intrapsychic background, as confirmed by other studies (74, 75), can be the basis for developing basic hope regarding the young person’s future, even in difficult, surprising, and extraordinary situations, as well as among the obstacles of everyday life.

The findings of the conducted study have significant practical implications, emphasizing the critical importance of emotional education for children and adolescents alike. Its primary aim should be to develop good stress management skills and build strong intrapsychic resources such as self-esteem and self-efficacy. Building a strong sense of self during the developmental stages promotes a psychological balance in challenging circumstances and serves as a universal resource in times of adversity (24). International organizations such as WHO and UNICEF (2, 3, 26) stress the necessity of implementing systemic solutions to support youth in these areas.

It is a challenge for all those present in the environment of adolescents aged 11–15, and at other stages of adolescence, to strengthen the internal resilience of the young generation by shaping the belief system of each individual into a positive attitude toward oneself and others, building a sense of reciprocated kindness, benevolence, and meaningfulness of the world, as well as personal effectiveness (70). The results of the presented study suggest a potential need for implementing intervention programs focused on supporting young people in building positive interpersonal relationships and enhancing their sense of security in the surrounding world. It is particularly important to address the specific needs of girls, who according to the findings, demonstrated greater vulnerability to the negative effects of the analyzed stressors.

This aligns with the developmental tasks of adolescence (21, 23) and the notions of youth resilience to stress and other known concepts of mental health maintenance through resource utilization (22, 62, 75, 76) in the face of situational and societal threats.

Moreover, there is a clear need to continue research in this field, given the ongoing dynamic changes in the broader social context. Longitudinal studies would be particularly valuable, enabling the observation of long-term effects of social stressors on adolescents’ mental health. Another important direction for further analysis would involve evaluating the effectiveness of various intervention strategies and their impact on improving the psychological well-being of young people.

## Data Availability

The raw data supporting the conclusions of this article will be made available by the authors, without undue reservation.
